# Exposure to an Indoor Cooking Fire and Risk of Trachoma in Children of Kongwa, Tanzania

**DOI:** 10.1371/journal.pntd.0003774

**Published:** 2015-06-05

**Authors:** Andrea I. Zambrano, Beatriz E. Muñoz, Harran Mkocha, Sheila K. West

**Affiliations:** 1 Wilmer Eye Institute, Johns Hopkins Hospital, Baltimore, Maryland, United States of America; 2 Kongwa Trachoma Project, Kongwa, Tanzania; London School of Hygiene & Tropical Medicine, UNITED KINGDOM

## Abstract

**Background:**

Elimination of blinding trachoma by 2020 can only be achieved if affected areas have effective control programs in place before the target date. Identifying risk factors for active disease that are amenable to intervention is important to successfully design such programs. Previous studies have linked sleeping by a cooking fire to trachoma in children, but not fully explored the mechanism and risks. We propose to determine the risk for active trachoma in children with exposure to cooking fires by severity of trachoma, adjusting for other known risk factors.

**Methods:**

Complete census of 52 communities in Kongwa, Tanzania, was conducted to collect basic household characteristics and demographic information on each family member. Information on exposure to indoor cooking fires while the mother was cooking and while sleeping for each child was collected. 6656 randomly selected children ages 1-9yrs were invited to a survey where both eyelids were graded for follicular (TF) and intense trachoma (TI) using the WHO simplified grading scheme. Ocular swab were taken to assess the presence of *Chlamydia trachomatis*.

**Findings:**

5240 (79%) of the invited children participated in the study. Overall prevalence for trachoma was 6·1%. Odds for trachoma and increased severity were higher in children sleeping without ventilation and a cooking fire in their room (TF OR = 1·81, 1·00–3·27 and TI OR 4·06, 1·96–8·42). Children with TF or TI who were exposed were more likely to have infection than children with TF or TI who were not exposed. There was no increased risk with exposure to a cooking fire while the mother was cooking.

**Conclusions:**

In addition to known risk factors for trachoma, sleeping by an indoor cooking fire in a room without ventilation was associated with active trachoma and appears to substantially increase the risk of intense inflammation.

## Author Summary

Trachoma remains the leading preventable cause of infectious blindness in the world. Identifying risk factors for active disease that are amenable to intervention is key to successfully designing effective control programs to eliminate blinding trachoma. Association between cooking fire and eye inflammation makes biological sense, and multiple other studies, but conducted in adults, have been reported. This is the first detailed study of the risk of trachoma or infection with *Chlamydia trachomatis* in children associated with a broader exposure to cooking fires. We were able to identify an important risk factor: a strong relationship between cooking fire exposure while sleeping and active trachoma that appears to substantially increase the risk of intense inflammation, which may play a role in perpetuating the disease in the community reservoir, the children. Hence a higher risk for transmission and re-emergence in communities seeking elimination. This finding may suggest modifications to current behavioral risks that were not considered before and may significantly impact the progression of the disease.

## Introduction

Trachoma remains the leading infectious cause of preventable blindness in the world, and a significant public health problem in endemic areas.[1] Elimination of trachoma by 2020 can only be achieved if all affected areas have effective control programs in place at a prudential period before the target date. The World Health Organization (WHO) advocates the SAFE strategy to control blinding trachoma (Surgery, Antibiotics, Facial cleanliness, and Environmental change) but identifying risk factors for trachoma that are amenable to intervention at family, or community level is important for designing successful control programs.[2]

Children are the main reservoir of the disease, so by controlling the risk factors and rate of infection in this population, we can decrease the community pools and risk of transmission, hence a great step in elimination of endemic trachoma. Known risk factors for active trachoma include young age, poor water access, unclean faces, and other household characteristics that are markers of poor socioeconomic status.[3] We had previously also found an association between children sleeping by a cooking fire and increased odds of trachoma; however the assessment of exposure was a simple question.[4] This association makes biological sense as multiple other studies, but conducted in adults, have associated pollution from cooking fires with eye irritation and alteration of ocular immunity. [5–14] However, no detailed study of the risk of trachoma or infection with *Chlamydia trachomatis* in children, associated with a broader exposure to sleeping rooms and rooms with a cooking fire has been carried out. We propose to determine the risk in children ages 1 to 9 years of active trachoma, both follicular diseases and intense trachoma, with a detailed assessment of exposure to indoor cooking fire.

## Materials and Methods

### Study Population

The research complied with the tenets of the Declaration of Helsinki and all guardians gave written informed consent for study procedures. Research was conducted with approval from the Johns Hopkins institutional review board and the national institute of medical research of Tanzania.

This cross-sectional study was conducted in 52 communities in Kongwa district of central Tanzania. These communities underwent mass drug administration for the previous five years, and at the time of the survey were at least one year from having had treatment with azithromycin. The school curriculum stresses face washing as one of its hygiene components but no other hygiene campaign has being carried out.

A complete census of households in each community was carried out and a random sample of 128 children from each community between ages 1–9 years was selected for the trachoma survey. A total of 5240 children from 4311 households were surveyed. Details are described below.

### Census

The census was carried out prior to the survey by a trained census team that collected demographic information for all household members and household information including type of roof of the house, presence of latrine, distance to water source, level of education of the household leader, number of children in the house, age and sex of the children. A specific cooking fire questionnaire was done to collect information about the type, use and location of cooking stoves/fires in and around the house, at different times of the year. The characteristics of the room where each resident child slept was directly observed and recorded. We observed the presence or not of a cooking fire, and observed whether the room had ventilation defined as present if either of the following were present: the room had windows that allowed air in or vents at the top of the walls (at least 3 inches of space between the roof and the wall where sky could be seen).

### Survey Visit and Trachoma Assessment

Examination of each everted eyelid was performed by trained trachoma grader using a 2·5X loupe. The trachoma grader was trained by a GTMP certified grader (HM), and had to have a kappa of >.6 against the trachoma certified grader. Trachoma was assessed in both eyes using the WHO simplified grading scheme, which assesses the presence or absence of follicular trachoma (TF), severe intense trachoma (TI), conjunctival scarring (TS), trichiasis (TT), and corneal opacity (CO). For this study, the relevant signs are TF and TI, signs of active trachoma.[15,16] For our analyses, we defined active trachoma as the presence TF or TI, alone or together, and Intense trachoma as TI alone, or with TF in at leat one eye.

For quality control purposes photographs of the right upper eyelid of a random 20% sample of children examined (using a Nikon D-40 camera with a 105mm f/2·8D AF Macro lens). These images were used to monitor the consistency of grading. Inter-observer agreement between the grader and the master grader (SW) was kappa 0·72 (95 CI: 0·62–0·82).

All children had ocular swabs taken from the left eye for determination of infection, using strict protocols to avoid field contamination. In each village a 5% sample of children had “air swabs” taken to check for field contamination. Samples were stored at KTP in a refrigerator and shipped within 30 days to the Johns Hopkins International Chlamydia Laboratory to be analyzed using the APTIMA ACT commercial test for *C*. *trachomatis* (Gen-Probe Inc., San Diego CA). Lab personnel were masked to the identified study and “air” swabs. None of the “air” swabs were positive.

### Data Analyses

For sleeping in a room with a cooking fire, we created an exposure index as follows: the lowest exposure was sleeping in a room without a cooking fire and with ventilation, the next lowest was sleeping in a room without a cooking fire but with no ventilation, the higher category was sleeping in a room with a cooking fire but the room had ventilation, and the highest exposure was sleeping in a room with a cooking fire and the room had no ventilation. However, few households were at the high extreme only 3% of children slept in a room with a cooking fire, regardless of ventilation. Therefore, we used three levels to reflect exposure: sleeping in a room with a cooking fire, sleeping in a room with no cooking fire and no ventilation, and sleeping in a room with no cooking fire and with ventilation.

Results were adjusted for age. Exposure while sleeping was assessed with logistic regression models to determine the associations between presence of TF and TI and exposure, adjusting for other risk factors. In the multivariate model, a backward elimination procedure was used to construct a parsimonious model that included only factors with significance level ≤ 0·5. Exposure during the time a mother cooked was also assessed. We used stratified analyses to evaluate the effect of exposure to a cooking fire on risk of infection in children with trachoma.

Random effects models including a random intercept for the community were used to account for the correlation of trachoma within residents of the same community. Less than 10% of the children belonged to the same household; in a sensitivity analysis we determined that adjusting for this level of clustering had no effects on our results. All analyses were conducted in SAS (version 9.2, SAS Institute Inc., Cary, NC, USA).

## Results

A total of 6656 children were randomly selected, and 1416 (21%) did not participate primarily because they were absent from the village the day of the exam; 5240 children from 4311 households were examined. Demographic characteristics of the participating children were slightly different than children who were non-participants (Table 1). Non- participants were more likely to be older, male, and live in houses where the water source was farther and the head of household had no former education. However, the two groups were similar in terms of the characteristics of the room where they slept. Non-participants tended to live in houses where the room with the cooking fire had ventilation. The majority, 99%, of households had an open fire stove with predominately wood or charcoal fuel.

**Table 1:** General characteristics of the sample by study participation.

Characteristic	Participants	Non Participants	p-value*
**Demographics**			
Number of children	5240	1416	
Age in years Mean (SD)	4·9 (2·5)	5·1 (2·5)	0·009
% Female	50·2	46·8	0·025
**Household**			
% No formal education of head of household	38·8	44·0	0·001
% More than 30 minutes from water source	55·2	59·5	0·008
% Roof made of mud/wood	11·6	12·2	0·60
% Has a latrine	78·6	77·5	0·35
**Cooking Fire Location and Ventilation**			
% Outside open air	1·6	1·9	0·002
% In a room with ventilation	39·4	43·7	
% In a room without ventilation	59·0	54·3	
**Exposure to cooking fire**			
% Child present in the room while cooking during the day	43·0	38·9	0·053
Child sleeps in a room			
% Without a cooking fire and ventilated	50·3	48·7	0·41
% Without a cooking fire and not ventilated	46·8	48·3	
% With a cooking fire	2·9	2·9	

* From a logistic model adjusting for age or a two sample t-test as appropriate

**Fig 1:**
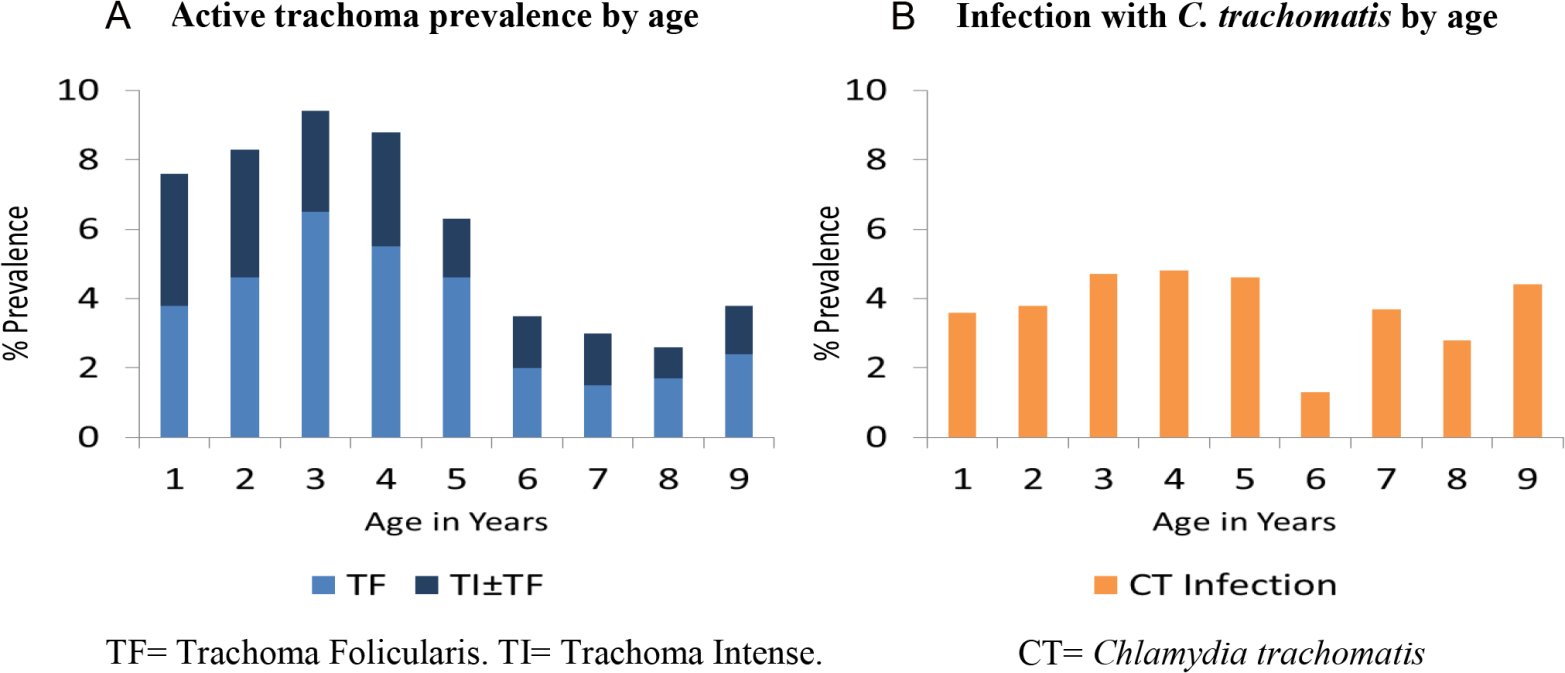
A) Active trachoma prevalence by age. B) Infection with *C*. *trachomatis* by age. **(**TF = Trachoma Folicularis. TI = Trachoma Intense. CT = *Chlamydia trachomatis)*.

The overall prevalence of active trachoma was 6·1%. Three year olds had the highest prevalence (9·5%). TI was present in all age groups and in 38% of the active trachoma cases, shown in Fig 1A. The overall *C*. *trachomatis* infection prevalence for these groups was 3.8% and prevalence by age is shown in Fig 1B.

A greater odds of active trachoma and severe trachoma was seen in children ages 1–5 years compared to the 6–9 year olds. After adjusting for age, a positive association was seen between active trachoma and severe trachoma and several indicators of socioeconomic status. Living in a household with no latrine, living farther from the water source, and in a house with a mud roof were all associated with active trachoma and severe trachoma (Table 2). Children whose head of household had no formal education were also significantly more likely to have active and severe trachoma. No increased risk was observed for children who spent time in the room with the cooking fire during cooking. However, we observed a dose response increase in odds of active trachoma, and especially severe trachoma, in children according to the proximity to the cooking fire and the degree of ventilation (Table 2). Compared to the lowest risk category, children who slept in a room without the cooking fire but no ventilation had an increased odds of trachoma, and children who slept in a room with a cooking fire had a 2·4 fold increased odds of active trachoma and a 5·5 fold increased odds of TI. in children who slept in a room with a cooking fire.

**Table 2:** Bivariate association of active trachoma/intense trachoma (age adjusted).

Characteristic	Active Trachoma (TF or TI)		Intense Trachoma (TI alone or with TF)	
	Odds Ratio	95% Confidence Interval	Odds Ratio	95% Confidence Interval
**Aged 1–5 vs. 6–9**	2·67	2·04–3·50	2·34	1·53–3·56
**Male gender**	1·07	0·85–1·34	0·97	0·67–1·39
**Unclean face (1–5 year old)**	1·87	1·42–2·46	3·12	1·93–5·07
**No formal education of household head**	1·56	1·24–1·96	1·96	1·36–2·82
**More than 30 minutes from water**	1·68	1·32–2·14	2·03	1·53–3·47
**House does not have a latrine**	1·34	1·04–1·75	1·72	1·17–2·53
**Mud roof vs. full/partial tin roof**	1·43	1·04–1·96	1·88	1·19–2·96
**Child present in the room while cooking during the day**	1·07	0·85–1·36	1·02	0·70–1·48
**Child sleeps in a room**				
**Without cooking fire & ventilation**	1·00		1·00	
**Without cooking fire & no ventilation**	1·43	1·12–1·82	1·77	1·18–2·65
**With cooking fire**	2·35	1·33–4·16	5·50	2·75–11·0

After multiple adjustments for age and the other variables, only longer distance to water remained a significant association with trachoma and severe trachoma. Living in a house with a mud roof, or no latrine, was no longer significant (Table 3). Children who slept in a room with a cooking fire were 1·8 times more likely to have active trachoma and 4·1 times as likely to have severe trachoma as children who slept in a room with ventilation and without a cooking fire.

**Table 3:** Multi-variate model of factors associated with active trachoma/intense trachoma.

Characteristic	Active Trachoma (TF or TI)		Intense Trachoma (TI)	
	Odds Ratio	95% Confidence Interval	Odds Ratio	95% Confidence Interval
**Aged 1–5 vs. 6–9**	2·73	2·04–3·65	2.42	1.55–3.79
**No formal education of household head**	1·41	1·10–1·81	1.64	1.10–2.42
**More than 30 minutes from water**	1·39	1·05–1·83	1.72	1.09–2.70
**Child sleeps in a room**				
Without a cooking fire & ventilation	1·00		1.00	
Without a cooking fire & no ventilation	1·33	1·03–1·71	1.57	1.04–2.38
With a cooking fire	1·81	1·00–3·27	4.06	1.96–8.42

Only the younger children were exposed to the cooking fire during the day while their mothers cooked, resulting in exposure confounded by age. We restricted the analyses to children age 0–5 years and examined the relationship between active and severe trachoma with exposure while cooking, according to the characteristics of the room (Table 4). Although the prevalence of active trachoma was highest in children who were exposed in a room with no ventilation, the test for trend was not significant.

**Table 4:** Active/intense trachoma in children aged 0–5 years with exposure to the cooking fire by being present in the room while mothers were cooking.

Cooking fire room	N	Active Trachoma		Intense Trachoma	
		%	p-value	%	p-value
**Outside open air**	26	3·9	0·51*	3·9	0·26
**Room with ventilation**	632	8·2		2·4	
**Room without ventilation**	985	8·7		3·7	

*Test for trend (Mantel-Haenszel Chi-Square)

We examined the infection rates in children according to trachoma status and type of sleeping room. We had few infections, but the trend for increasing infection in children with trachoma who slept in unventilated rooms or slept with a cooking fire was observed (Table 5).

**Table 5:** *C*. *trachomatis* infection in children by type of sleeping room and active trachoma status.

Type of sleeping room	Active Trachoma	N	% positive for *C*.*T*. Infection
**Without a cooking fire and ventilated**	Absent	2341	1·4
	Present	125	34·4
**Without a cooking fire and not ventilated**	Absent	2136	1·8
	Present	160	36·3
**With a cooking fire**	Absent	127	1·6
	Present	15	46·7

## Discussion

Our study found a strong relationship between cooking fire exposure while sleeping and active trachoma, especially severe trachoma in children.

There is good biologic plausibility why trachoma may be higher in children exposed to indoor air pollution (IAP). In addition to increased tearing and irritation, which may result in auto-re-infection, IAP appears to have a direct effect on the immune system. In women who were users of biomass fuels, an increased TH2 response was described with an increase in the Treg cells, CD4+ and CD25+, a subset that can inhibit effector T cell response.[9] In this case, much of the regulatory activity is exerted by IL-10 and TGFβ, perpetuating the TH2 response and leading to chronic inflammation, and less clearance of infection.[7,8] Our data supports this finding, as we noted that infection rates were higher with TF in children who were exposed to a cooking fire, suggesting a prolongation of infection. Another study in children described the effects in T cell immunity from exposure to ambient polycyclic aromatic hydrocarbons, which are compounds produced from combustion of organic matter such as wood or coal. Epigenetic modifications associated with impaired immunity suggesting an increased TH2 response was observed as well.[17] For clearance of acute trachoma, an adequate CD4+ response of the Th1 phenotype appears to be necessary, and the Th1 cytokine gamma interferon assists in infection clearance. So not only might IAP lead to effects that delay clearance of infection, but might also increase risk for trachoma sequelae.

Previous studies of exposure to a cooking fire while cooking in women in non-trachoma areas reported an increasing eye irritation, conjunctival damage and tearing of the eyes.[5,6,18] Similar ocular effects were reported in children exposed to wildfire smoke exposure in California Ellegard and Diaz describe in their studies an increase in tear production or “tears while cooking” (TWC) in women and the direct association with indoor air pollution (IAP). The study also found a strong relationship between ocular symptoms in women who cooked inside a room, compared to women who cooked outside, suggesting a role for ventilation.[6] We did not find a statistically significant association of trachoma in children who were in the cooking fire room during cooking, although the greatest prevalence of trachoma in children age five years and under was in those in a cooking room with no ventilation.

In addition, the type of stove used also has been shown to play a role in eye symptoms in women. Smoke free stove versus traditional or open fire stoves have been assessed in relationship with the amount of IAP in the house and symptoms that the women experienced.[5,6] Women were found to have increased eye symptoms when exposed to open fire stoves especially those who use wood or charcoal as fuel. In our study population 99% of the women cooked using an open fire stove with predominately wood or charcoal, adding to the burden of indoor air pollution.

It is possible that the children who sleep in a room with cooking fire or rooms that are not ventilated belong to poorer families with less access to water and general sanitation. We tried to account for these socioeconomic factors in our study and found a strong, independent effect of sleeping in a room with a cooking fire.

Interestingly, we found that 3% of the children slept in a room with a cooking fire, compared to the results from the same district in 1986, where 60% of the children slept in a room with a cooking fire.[4] In 1986, the estimated prevalence of trachoma in children ages 1–7 years was 60%, and at that time, most of the children slept on animal skins in the room with a cooking fire. Since 1986, many changes have occurred, with most households now building a separate cooking fire area from the sleeping room, and using beds for sleeping. Trachoma has declined in this district, most markedly in the last eight years in connection with trachoma control measures, to an overall estimate last year of 12%. This change is encouraging, suggesting some environmental and socioeconomic improvement in these communities.

A difference between age groups was noted as well, where younger children had more TF and TI than older children. This age difference in clinical signs has previously been described.[19–27] and may reflect decreased exposure to re-infection as children reach school age. Of note, the older aged children did not spend time during the day in the room with the cooking fire, and we had to confine our analyses of exposure during cooking to the younger aged children.

There were some significant differences observed between the participating and non-participating children, the latter being older and more likely to be male, and to live in a house farther from a water source. However, the groups were similar in terms of exposure to cooking fire while sleeping. Since those who are older are less likely to have trachoma, but those who live far from water are more likely to have trachoma, the effects of these differences are uncertain. In any case, there was no difference in exposure to cooking fire, suggesting an absence of bias due to differential participation.

In addition, these communities are also undergoing mass drug administration. The last MDA was more than one year ago for these villages, but the low rates of trachoma, 6%, likely reflect at least in part the high compliance with this program. We were underpowered to detect a significant difference in infection rates in trachoma in children who slept in a room with a cooking fire and those who did not, although the trend was observed. The fact that Taylor et al in 1986 found a similar risk for sleeping next to a cooking fire in these communities prior to any intervention argues for an effect of exposure on trachoma even when disease rates are low or in the presence of a program with MDA.[4]

In conclusion we confirmed that those children who sleep in a room with a cooking fire have an increased risk of trachoma independently from other risk factors for disease. The prevalence of exposure to sleeping next to a cooking fire in this population has declined over time, and programs to encourage continued decline are warranted. Further studies on the impact of exposure to indoor air pollution and the risk of trachoma sequelae should be done.

## Supporting Information

S1 Checklist STROBE checklist. (DOCX)
